# Measuring medication adherence in patients with incident hypertension: a retrospective cohort study

**DOI:** 10.1186/s12913-017-2073-y

**Published:** 2017-02-13

**Authors:** Karen L. Tang, Hude Quan, Doreen M. Rabi

**Affiliations:** 1 Department of Medicine, University of Calgary, 3330 Hospital Drive NW, T2N 4N1 Calgary, Alberta Canada; 2 Department of Community Health Sciences, University of Calgary, 3280 Hospital Drive NW, T2N 4Z6 Calgary, Alberta Canada

**Keywords:** Drug Utilization, Prescription Drugs, Adherence, Claims data, Hypertension

## Abstract

**Background:**

Though pharmacy claims data are commonly used to study medication adherence, there remains no standard operational definition for adherence especially for patients on multiple medications. Even when studies use the same terminology, the actual methods of calculating adherence can differ drastically. It is unclear whether the use of different definitions results in different conclusions regarding adherence and associated outcomes. The objective of our study was to compare adherence rates and associations with mortality using different operational definitions of adherence, and using various methods of handling concurrent medication use.

**Methods:**

We conducted a cohort study of patients aged ≥65 years from Manitoba, Canada, with incident hypertension diagnosed in 2004 and followed to 2009. We calculated adherence rates to anti-hypertensive medications using different operational definitions of medication adherence (including interval and prescription based medication possession ratios [MPR] and proportion of days covered [PDC]). For those on concurrent medications, we calculated adherence rates using the different methods of handling concurrent medication use, for each definition. We used logistic regression to determine the association between adherence and mortality for each operational definition.

**Results:**

Among 2199 patients, 24.1% to 90.5% and 71.2% to 92.7% were considered adherent when using fixed interval and prescription-based interval medication possession ratios [MPRi and MPRp] respectively, depending on how concurrent medications were handled. Adherence was inversely associated with death, with the strongest association for MPRp measures. This association was significant only when considering adherence to any anti-hypertensive [aOR 0.70, 95% CI 0.51, 0.97], or when the mean of the class-specific MPRp’s [adjusted OR 0.71, 95% CI 0.53, 0.95] was used. No significant association existed when the highest or lowest class-specific MPRp was used as the adherence estimate.

**Conclusion:**

The range of adherence estimates varies widely depending on the operational definition used. Given less variation in adherence rates and their stronger association against mortality, we recommend using prescription-based MPR’s to define medication adherence.

## Background

Medication adherence is defined as the extent to which a patient takes prescribed medications according to the dosage and frequency recommended by the provider [1, 2]. Non-adherence to prescribed medications is associated with poor treatment outcomes, increased hospitalizations, and increased cost to the health care system for chronic diseases such as hypertension, diabetes, and heart failure [3–5]. Despite the presence of a clinical definition of medication adherence, there remains no standard operational definition for medication adherence in health research, especially when using pharmacy claims data [6].

In the literature, there are a wide number of terms and operational definitions used to assess medication adherence (see Table 1 for a list of definitions used in the hypertension literature). Even when studies use the same terms, the operationalized or practical definition presented, the time frame considered, and the method of calculating adherence often differ. Most studies use the “medication possession ratio” (MPR) [1, 7] or similar related adherence measures, generally defined as the proportion of a time period where a medication supply is available [1]. Variations in MPR calculations stem from different denominators used, which can either be a fixed time interval or a variable period between prescriptions. The former is termed “interval based MPR” [MPRi] and the latter, “prescription based MPR” [MPRp] [8]. MPR can be reported as a continuous or as a dichotomized measure, where patients with an MPR above a certain threshold are considered “adherent”. While the level of optimal adherence may differ for different clinical conditions, a threshold of 0.80 [1] is conventionally used.

**Table 1:** Terminology and operational definitions of medication adherence used in hypertension literature

Terminology	Operational Definition	Time frame	Thresholds to dichotomize adherent versus non-adherent
Continuous multiple –interval gap (CMG) [24–26]	• “Percentage of days that a patient did not possess medication” [24, 26] • $\frac{{\mathrm{Total}\ \mathrm{number}\ \mathrm{of}\ \mathrm{days}\ \mathrm{without}\ \mathrm{medications}\ \mathrm{between}\ \mathrm{first}\ \mathrm{and}\ \mathrm{last}\ \mathrm{fills}}^{\mathrm{a}}}{\mathrm{Number}\ \mathrm{of}\ \mathrm{days}\ \mathrm{between}\ \mathrm{first}\ \mathrm{and}\ \mathrm{last}\ \mathrm{pharmacy}\ \mathrm{fills}}$ [25]	• 1 year [24, 26]	• <20% or <0.20 [24, 25] • Division into 3 groups [26]: <20%, 20% to 59%, and ≥60%
Continuous single- interval medication availability (CSA) [25]	• $\frac{\mathrm{Days}\ \mathrm{supply}\ \mathrm{a}\mathrm{t}\ \mathrm{a}\ \mathrm{single}\ \mathrm{pharmacy}\ \mathrm{fill}}{\mathrm{Number}\ \mathrm{of}\ \mathrm{days}\ \mathrm{before}\ \mathrm{t}\mathrm{he}\ \mathrm{next}\ \mathrm{pharmacy}\ \mathrm{fill}\ \mathrm{for}\ \mathrm{t}\mathrm{he}\ \mathrm{same}\ \mathrm{medication}}$ [25]	• 1 year [25]	• ≥0.80 [25]
Medication Possession Ratio (MPR) [13, 25, 27–33], *or* Medication Refill Adherence [34]	• $\frac{\mathrm{Total}\ \mathrm{days}\ \mathrm{supply}\ \mathrm{from}\ {1}^{\mathrm{st}}\ \mathrm{fill}\ \mathrm{date}\ \mathrm{to}\ \mathrm{last}\ \mathrm{fill}\ {\mathrm{date}}^{\mathrm{a}}}{\mathrm{Days}\ \mathrm{between}\ \mathrm{first}\ \mathrm{fill}\ \mathrm{date}\ \mathrm{to}\ \mathrm{last}\ \mathrm{fill}\ \mathrm{date}}$ [25, 27, 29, 30, 34]	• 6 months [27, 33]	• No dichotomization- Presented as a quantitative measure [33]
	• $\frac{\mathrm{Total}\ \mathrm{days}\ \mathrm{supply}\ \mathrm{between}\ {1}^{\mathrm{st}}\ \mathrm{fill}\ \mathrm{date}\ \mathrm{to}\ \mathrm{last}\ \mathrm{fill}\ \mathrm{date}}{\mathrm{Days}\ \mathrm{in}\ \mathrm{the}\ \mathrm{study}\ \mathrm{period}}$ [28, 32]	• 9 months [34]	• ≥80% or ≥ 0.80 [13, 25, 27, 29, 30, 34, 35]
	• $\frac{\mathrm{Total}\ \mathrm{days}\ \mathrm{supply}\ \mathrm{between}\ {1}^{\mathrm{st}}\ \mathrm{fill}\ \mathrm{date}\ \mathrm{to}\ \mathrm{last}\ \mathrm{fill}\ \mathrm{date}}{\mathrm{Fixed}\ \mathrm{observation}\ \mathrm{period}\ \left(\mathrm{e}.\mathrm{g}.\ 1\ \mathrm{year}\right)}$ [35]	• 1 year [27, 31, 32, 35]	• Division into 3 groups [28, 32]: 80–100%: High adherence 50–79%: Moderate adherence <50%: Low adherence
	• $\frac{\mathrm{Total}\ \mathrm{days}\ \mathrm{supply}\ \mathrm{between}\ {1}^{\mathrm{st}}\ \mathrm{fill}\ \mathrm{date}\ \mathrm{to}\ \mathrm{last}\ \mathrm{fill}\ \mathrm{date}\ }{\mathrm{Fixed}\ \mathrm{observation}\ \mathrm{period}\ \left(\mathrm{e}.\mathrm{g}.\ 1\ \mathrm{year}\right)\ }$ [31]	• 3 years [28]	
	• $\frac{\mathrm{Number}\ \mathrm{of}\ \mathrm{days}\ \mathrm{with}\ \mathrm{medication}}{\mathrm{Days}\ \mathrm{in}\ \mathrm{study}\ \mathrm{period}}$ [13]	• Up to 4 years [13]	
	• $\frac{\frac{\mathrm{Number}\ \mathrm{of}\ \mathrm{tablets}\ \mathrm{dispensed}}{\mathrm{Daily}\ \mathrm{dose}}\times \left(\mathrm{Number}\ \mathrm{of}\ \mathrm{dispensing}\mathrm{s}-1\right)}{\mathrm{Number}\ \mathrm{of}\ \mathrm{days}\ \mathrm{between}\ \mathrm{last}\ \mathrm{and}\ \mathrm{first}\ \mathrm{dispensing}}$ [33]	• Up to 6 years [30]	
Proportion of Days Covered (PDC) [36, 37]	• “The proportion of days covered by any anti-hypertensive medication… determined based on number of days supplied and quantity of medication dispensed for each prescription” [37]	• 3 months [37]	• ≥0.80 [36, 37]
	• $\frac{\mathrm{Number}\ \mathrm{of}\ \mathrm{days}\ \mathrm{with}\ \mathrm{medication}}{\mathrm{Days}\ \mathrm{in}\ \mathrm{study}\ \mathrm{period}}$ [36]	• 1 year [36]	
MedsIndex [33]	• “Score out of 100 calculated from prescription refill history” [33] • $\frac{\mathrm{Days}\ \mathrm{supply}\ \mathrm{a}\mathrm{t}\ \mathrm{a}\ \mathrm{single}\ \mathrm{pharmacy}\ \mathrm{fill}}{\mathrm{Number}\ \mathrm{of}\ \mathrm{a}\mathrm{ctual}\ \mathrm{days}\ \mathrm{between}\ \mathrm{repeats}}$ [38]	• 6 months [33]	• No dichotomization- Presented as a quantitative measure [33]

Despite wide acceptance and usage of MPR, there remains significant variation not only in its specific calculation as mentioned above, but also in methods of managing multiple concurrent medications (“polytherapy”) to obtain a single measure of adherence for each patient (see Table 2). Methods of calculating an MPR estimate for patients on polytherapy can consider either adherence to individual drug classes or adherence overall to any medication. In addition, a related measure to MPR termed Proportion of Days Covered [PDC] is often preferred in polytherapy due to its lower risk of overestimation [9]; PDC is defined as the proportion of days in a fixed observation period where at least one of multiple medications is available.

**Table 2:** Methods of handling use of multiple concurrent anti-hypertensive medications when measuring adherence in hypertension literature

Measure	Method of handling concurrent medications	Method considers adherence to *any* medication versus adherence to *each* medication
CMG [24–26]	Sum of the total number of gap days (days the patient did not possess the medication) for each anti-hypertensive medication divided by the sum of the total number of days the patient was prescribed each anti-hypertensive medication [24, 26]	Any
	Calculate and report CMG for each anti-hypertensive medication class [26]	Each
	Calculate CMG for each anti-hypertensive medication class, then take the mean CMG for each patient [25]	Each
CSA [25]	Calculate CSA for each anti-hypertensive medication class, then take the mean CSA for each patient [25]	Each
MPR [13, 25, 27–35] [39]	Exclude patients not on monotherapy [28]	N/A
	Method of handling polytherapy not reported [33]	N/A
	Calculate and report for each class of anti-hypertensive medication [27, 30]	Each
	Take the single medication with the maximum number of days supply over one year period and use this single medication for the MPR estimate for the patient [35]	Each
	Calculate MPR for each anti-hypertensive mediation class, then take the lowest MPR (for the medication with lowest adherence) as the MPR estimate for each patient [34, 39]	Each
	Calculate MPR for each anti-hypertensive medication class, then take the mean MPR for each patient [25, 29, 32, 34]	Each
	Include all days where at least one anti-hypertensive medication was available in the numerator of the ratio of number of days with medication to days in study period [13]	Any
	Calculate numerator for MPR by taking sum of all days supply of all anti-hypertensive medications prescribed as the numerator [40]	Any
	Include only days where all anti-hypertensive medications of interest were available [31]	Each
PDC [36, 37]	Include all days where at least one anti-hypertensive medication was available in the numerator of the ratio of number of days with medication to days in study period [36, 37]	Any
MedsIndex [33]	Method of handling polytherapy not reported [33]	N/A

*Abbreviations*: *CMG* Continuous multiple-interval gap, *CSA* Continuous single-interval medication availability, *MPR* Medication possession ratio, *PDC* proportion of days covered, *N/A* Not applicable

The many different operational definitions and methods used to handle polytherapy may result in very different adherence estimates and highly variable conclusions [8], highlighting the need to harmonize operational definitions of adherence used in health research. Our study objective was to compare adherence rates using the different operational definitions (MPRi, MPRp, and PDC) and methods of handling concurrent medications, and to determine the strengths of association between medication adherence using these different operational definitions with all-cause mortality in a population of Canadians with newly diagnosed hypertension.

## Methods

### Sources of data

Administrative data extracted from hospital discharge abstracts and physician claims were collected in Manitoba, Canada from April 1, 1997 to March 31, 2009. We linked this database with vital statistics for mortality data, the health insurance registry for demographic data, and the Manitoba Pharmacare prescription database for drug data. Drug data from April 1, 2002 to March 31, 2009 were used for this study. Given the universality of Manitoba Pharmacare, this database contains dispensing records for all outpatient prescription drug claims for all Manitoban residents, with the exception of First Nations and Inuit persons, inmates incarcerated in federal penitentiaries, military personnel, RCMP, and veterans; these subgroups are covered by the federal drug benefit plan [10]. Institutional ethics board approval was obtained from the University of Calgary Conjoint Health Research Ethics Board. Manitoba Health granted access to the administrative databases used in this study. Direct patient consent was not obtained, as the data being analyzed were routinely collected administrative health data that had been de-identified prior to receipt of data.

### Study population

The 25 diagnosis fields in hospital discharge abstracts and the 1 field in physician claims were searched for the relevant International Classification of Diseases, Ninth and Tenth Revisions (ICD-9 and ICD-10) codes for hypertension (401.x-405.x, 110.x-115.x). The case definition for incident hypertension in the databases was one hospitalization or two physician claims within 2 years with a hypertension ICD code. We determined incidence with a three-year washout period [11]. Index date of diagnosis was the first date at which the case definition was fulfilled. Patients without at least a single prescription refill within one year after the first prescription fill in any of the five antihypertensive medication classes of interest (thiazide-type diuretics, beta blockers [BB], calcium channel blockers [CCB], angiotensin converting enzyme inhibitors or angiotensin receptor blockers [ACEI/ARB], or a combination containing at least one of the above classes; see Appendix 1), and patients who died within one year of the first prescription fill were excluded to ensure that each operational definition could be calculated for each patient. The study population included a random sample of Manitoba residents aged ≥65 years with incident hypertension, with an index date of diagnosis between April 1, 2004 and March 31, 2005.

### Sample size calculation

Using conservative assumptions, the target sample size, assuming that the mortality rate of patients over the age of 65 years old with hypertension who are non-adherent to medications is 17% [12], to achieve a power of 80%, a level of significance of 5%, and to detect a relative risk ratio of mortality of 0.72 [13] for patients who are adherent to medications compared with patients who are non-adherent to medications, is 2124. To account for the exclusion of approximately 30% of the entire sample either because they have not had had a single fill or refill of any anti-hypertensive medications in the entire follow-up period, or die within the first year of diagnosis, 3000 patients with incident hypertension were randomly selected for analysis. Random sampling was performed using the probability proportional to size method based on age, sex, and comorbidities.

### Measures of medication adherence

We calculated MPR using the following 2 formulae (interval-based and prescription-based MPR respectively) for an observation period of 1 year:

$$ \mathrm{MPRi}=\frac{\mathrm{Days}\ \mathrm{supply}\ \mathrm{of}\ \mathrm{medication}}{365\ \mathrm{days}},\mathrm{capped}\ \mathrm{at}\ 1 $$

$$ \mathrm{MPRp}=\frac{\mathrm{Days}\ \mathrm{supply}\ \mathrm{of}\ \mathrm{medication}\ \mathrm{excluding}\ \mathrm{supply}\ \mathrm{from}\ \mathrm{last}\ \mathrm{refill}}{\mathrm{Last}\ \mathrm{refill}\ \mathrm{date}\hbox{-} \mathrm{First}\ \mathrm{refill}\ \mathrm{date}},\ \mathrm{capped}\ \mathrm{at}\ 1 $$

Medications within the same medication class (Appendix 1) were considered interchangeable.

For patients on polytherapy in our study, we calculated four different MPRi’s and MPRp’s: a) MPR considering adherence to *any* antihypertensive, obtained by first summing the days supply of each anti-hypertensive prescription in the numerator [9], then dividing by the denominator as stated above; b) average of the MPR’s specific to each anti-hypertensive medication class [8, 14]; c) calculating the MPR’s specific to each medication class, then taking the highest of the class-specific MPR’s [15]; and d) calculating the MPR’s specific to each medication class, then taking the lowest of the class-specific MPR’s [16]. We termed these methods “sum MPR”, “mean MPR”, “high MPR” and “low MPR” respectively. Of note, for sum MPR, no medication class-specific MPRs are calculated. The denominator used for sum MPRp is the last refill date for any of the medication classes minus the first refill date for any of the medication classes (even if this medication class is not the same one used for the last refill date). We also calculated PDC for each patient by dividing the number of days where at least one medication was available (each day is considered individually and is a binary measure) [9] by 365 days.

MPR’s and PDC’s were additionally dichotomized using the standard threshold of 0.80. For patients on anti-hypertensive therapy prior to the index date, adherence measures were calculated from the first prescription fill starting from 2002. If prescription refills were obtained prior to exhaustion of the supply of the previous fill for the same medication class, the date of the refill was prorated to start on the day after exhaustion of the previous supply.

### Mortality

The primary outcome measure was mortality from the period 2005 to 2009 (a maximum of 5 years of follow-up). Any patients moving out-of-province or reaching the end of the observation period were censored.

### Statistical analysis

Means and medians for continuous medication adherence variables were reported. These variables were also dichotomized, and Fisher’s exact test was used to compare the proportion of adherent and non-adherent patients who had died. We also stratified this analysis by age, sex, income, and number of comorbidities. Multiple logistic regression models were employed to model the odds of death for those who are adherent to medications, compared to those who were non-adherent to medications, using the different operational definitions for adherence. The threshold used to define adherence was an MPR or PDC ≥ 0.80. Unadjusted and adjusted Cox proportional hazards regressions were performed for each operational definition of adherence, to assess time to death. Given their potential to confound the association between medication adherence and mortality, we controlled for demographic and comorbidity variables in our adjusted logistic regression and Cox proportional hazards regression models; these variables included age, sex, Charlson comorbidity index, income quintile, and previous hospitalization for cardiovascular disease within three years prior to the index date of hypertension diagnosis. Data on health-related behaviours, such as diet and activity levels, were not available given the nature of data used in this study and thus no adjustment for these variables were undertaken. However, previous studies have shown weak correlations and non-statistically significant associations between health-related behaviours and medication adherence [17–20]. Health-related behaviours are therefore unlikely to be a significant confounder in the associations between medication adherence and mortality. All analyses were conducted using SAS Version 9.4 (SAS Institute, Cary, NC).

### Sensitivity analysis

Because there is no robust evidence base to suggest that an MPR or PDC threshold of 0.80 used to classify those who are adherent versus non-adherent to medications is optimal or superior to other thresholds, we conducted sensitivity analysis, using various other adherence thresholds. For this sensitivity analysis, adjusted logistic regression as described above was also performed for thresholds of 0.70, 0.75, 0.85, and 0.90, for each of the operational definitions studied.

## Results

### Baseline characteristics

From a total of 5189 eligible patients aged 65 years or older with incident hypertension diagnosed in the 2004 fiscal year, a random sample of 3000 patients were selected for analysis. Of this sample of 3000 patients, we excluded those with no prescription filled for any anti-hypertensive in the entire follow-up period (*n* = 652), those who died within one year of their first anti-hypertensive prescription fill (*n* = 51), and those without at least one medication refill within one year of their first prescription fill (*n* = 98). Our final cohort comprised of 2199 patients. The median and mean follow-up durations were 4.41 years (interquartile range 4.11, 4.72) and 4.13 years (standard deviation 0.99) respectively.

Baseline characteristics can be found in Table 3. Overall, mean (SD) age was 75.2 (7.0) years, 45.4% were male, 12.2% had a previous hospitalization for cardiovascular disease, 33.7% were new users of anti-hypertensives, and 64.7% were on monotherapy. Comparison of baseline characteristics between non-adherent and adherent groups depended on the adherence measure being used (Table 3).

**Table 3:** Demographics and descriptive characteristics of medication use, for adherent versus non-adherent users as defined by three different operational definitions

		Mean MPRp			Mean MPRi			PDC		
		Non-adherent *n* = 479	Adherent *n* = 1716	*P* value	Non-adherent *n* = 916	Adherent *n* = 1283	*P* value	Non-adherent *n* = 533	Adherent *n* = 1666	*P* value
Age	Mean (SD)	74.7 (6.7)	75.1 (7.1)	0.093	74.9 (6.9)	75.4 (7.1)	0.084	74.7 (6.8)	75.3 (7.1)	0.127
	65-69 years	65-69 years 143 (29.9%)	65–69 years 143 (29.9%) 488 (28.4%	0.287	272 (29.7%)	359 (28.0%)	0.556	161 (30.2%)	470 (28.2%)	0.589
	70–74 years	131 (27.4%)	423 (24.7%)		240 (26.2%)	315 (24.6%)		140 (26.3%)	415 (24.9%)	
	75–79 years	96 (20.0%)	365 (21.3%)		191 (20.9%)	273 (21.3%)		113 (21.2%)	351 (21.1%)	
	80–84 years	71 (14.8%)	252 (14.7%)		126 (13.8%)	197 (15.4%)		71 (13.3%)	252 (15.1%)	
	85+ years	38 (7.9%)	188 (11.0%)		87 (9.5%)	139 (10.8%)		48 (9.0%)	178 (10.7%)	
Sex	Male	Male 225 (47.0%)	773 (45.1%)	0.468	418 (45.6%)	581 (45.3%)	0.896	266 (49.9%)	733 (44.0%)	0.019
Urban/Rural	Urban	271 (56.6%)	1013 (59.0%)	0.299	520 (56.8%)	768 (59.9%)	0.204	296 (55.5%)	992 (59.5%)	0.253
	Rural	200 (41.8%)	664 (38.7%)		379 (41.4%)	485 (37.8%)		225 (42.2%)	639 (38.4%)	
	Missing	8 (1.7%)	39 (2.3%)		17 (1.9%)	30 (2.3%)		12 (2.3%)	35 (2.1%)	
Income quintile	1 (Lowest)	100 (20.9%)	332 (19.4%)	0.284	169 (18.5%)	264 (20.6%)	0.283	108 (20.3%)	325 (19.5%)	0.031
	2	135 (28.2%)	384 (22.4%)		237 (25.9%)	282 (22.0%)		151 (28.3%)	360 (22.1%)	
	3	98 (20.5%)	367 (21.4%)		194 (21.2%)	272 (21.2%)		106 (19.9%)	360 (21.6%)	
	4	73 (15.2%)	301 (17.5%)		147 (16.1%)	228 (17.8%)		73 (13.7%)	302 (18.1%)	
	5	65 (13.6%)	293 (17.1%)		152 (16.6%)	207 (16.1%)		83 (15.6%)	276 (16.6%)	
	Missing	8 (1.7%)	39 (2.3%)		17 (1.9%)	30 (2.3%)		12 (2.3%)	35 (2.1%)	
Comorbidities	MI	19 (4.0%)	77 (4.5%)	0.705	39 (4.3%)	48 (4.5%)	0.833	22 (4.1%)	75 (4.5%)	0.809
	CHF	24 (5.0%)	97 (5.7%)	0.651	45 (4.9%)	76 (5.9%)	0.343	30 (5.6%)	91 (5.5%)	0.913
	CVA	23 (4.8%)	72 (4.2%)	0.611	42 (4.6%)	53 (4.1%)	0.671	23 (4.3%)	72 (4.3%)	1.000
	Dementia	5 (1.0%)	31 (1.8%)	0.311	9 (1.0%)	28 (2.2%)	0.042	7 (1.3%)	30 (1.8%)	0.563
	Renal Failure	7 (1.5%)	26 (1.5%)	1.000	13 (1.4%)	20 (1.6%)	0.860	9 (1.7%)	24 (1.4%)	0.684
Charlson Comorbidities	0	262 (54.7%)	930 (54.2%)	0.760	508 (55.5%)	685 (53.4%)	0.521	286 (53.7%)	907 (54.4%)	0.203
	1	169 (35.3%)	629 (36.7%)		321 (35.0%)	480 (37.4%)		187 (35.1%)	614 (36.9%)	
	2+	48 (10.0%)	157 (9.2%)		87 (9.5%)	118 (9.2%)		60 (11.3%)	145 (8.7%)	
New Users		144 (30.1%)	594 (34.6%)	0.063	325 (35.5%)	415 (32.4%)	0.131	200 (37.5%)	540 (32.4%)	540 (32.4%)
Monotherapy		332 (69.3%)	1090 (63.5%)	0.020	401 (43.8%)	1021 (79.6%)	<0.001	401 (75.2%)	1021 (61.3%)	<0.001
Maximum Number of Concurrent Medications	1	175 (36.5%)	673 (39.2%)	0.649	259 (28.3%)	589 (45.9%)	<0.001	241 (45.2%)	607 (36.4%)	<0.001
	2	194 (40.5%)	627 (36.5%)		384 (41.9%)	439 (34.2%)		205 (38.5%)	618 (37.1%)	
	3	83 (17.3%)	315 (18.4%)		204 (22.3%)	195 (15.2%)		69 (13.0%)	69 (13.0%)	
	4	26 (5.4%)	90 (5.2%)		66 (7.2%)	51 (4.0%)		18 (3.4%)	99 (5.9%)	
	5	1 (0.2%)	9 (0.5%)		3 (0.3%)	7 (0.6%)		0 (0.0%)	10 (0.6%)	
	6	0 (0.0%)	2 (0.1%)		0 (0.0%)	2 (0.2%)		0 (0.0%)	2 (0.1%)	

*Abbreviations*: *SD* standard deviation, *MI* myocardial infarction, *CHF* congestive heart failure, *CVA* cerebrovascular accident, CVD cardiovascular disease

### Adherence rates by operational definition

For patients on monotherapy (*n* = 1422), MPRi adherence estimates (mean 0.83, SD 0.23) were similar though consistently lower than the equivalent MPRp estimates (mean 0.87, SD 0.19, see Table 4). The PDC and MPRi methods gave identical adherence estimates for those on monotherapy only. These findings were consistent even with stratification by age, sex, income, and comorbidities.

**Table 4:** Mean and median adherence rates, and proportion with adherence ≥0.80 for patients on monotherapy (*n* = 1422)

			Range	Mean (SD)	Median (IQR)	Proportion ≥0.80
Overall		MPRi	0.04–1.00	0.83 (0.23)	0.95 (0.75, 0.99)	71.8%
		MPRp	0.04–1.00	0.87 (0.19)	0.96 (0.82, 1.00)	76.7%
		PDC	0.08–1.00	0.82 (0.23)	0.93 (0.72, 0.99)	67.9%
Age	65 to 74 years	MPRi	0.08–1.00	0.82 (0.23)	0.93 (0.72, 0.99)	67.9%
		MPRp	0.09–1.00	0.85 (0.21)	0.95 (0.78, 1.00)	73.6%
		PDC	0.08–1.00	0.82 (0.23)	0.93 (0.72, 0.99)	67.9%
	75 years and over	MPRi	0.04–1.00	0.84 (0.22)	0.95 (0.77, 0.99)	73.4%
		MPRp	0.04–1.00	0.87 (0.19)	0.96 (0.83, 1.00)	77.9%
		PDC	0.04–1.00	0.84 (0.22)	0.95 (0.77, 0.99)	73.4%
Sex	Male	MPRi	0.04–1.00	0.82 (0.23)	0.94 (0.73, 0.99)	67.9%
		MPRp	0.04–1.00	0.85 (0.21)	0.95 (0.80, 1.00)	73.6%
		PDC	0.04–1.00	0.82 (0.23)	0.94 (0.73, 0.99)	67.9%
	Female	MPRi	0.10–1.00	0.84 (0.22)	0.96 (0.78, 0.99)	73.4%
		MPRp	0.08–1.00	0.87 (0.19)	0.96 (0.82, 1.00)	77.9%
		PDC	0.10–1.00	0.84 (0.22)	0.96 (0.78, 0.99)	73.4%
Income	Lowest quintile	MPRi	0.05–1.00	0.83 (0.24)	0.95 (0.77, 0.99)	73.1%
		MPRp	0.04–1.00	0.87 (0.20)	0.96 (0.83, 1.00)	77.9%
		PDC	0.05–1.00	0.83 (0.24)	0.95 (0.77, 0.99)	73.1%
	Highest quintile	MPRi	0.10–1.00	0.85 (0.20)	0.95 (0.74, 0.99)	71.7%
Charlson comorbidities	0 or 1 comorbidities	MPRi	0.04–1.00	0.84 (0.22)	0.95 (0.76, 0.99)	72.6%
		MPRp	0.08–1.00	0.87 (0.19)	0.96 (0.82, 1.00)	77.2%
		PDC	0.04–1.00	0.84 (0.22)	0.95 (0.76, 0.99)	72.6%
	2+ comorbidities	MPRi	0.05–1.00	0.79 (0.26)	0.92 (0.61, 1.00)	63.9%
		MPRp	0.04–1.00	0.83 (0.23)	0.94 (0.77, 1.00)	71.5%
		PDC	0.05–1.00	0.79 (0.26)	0.92 (0.61, 1.00)	63.9%

*Abbreviations: SD* standard deviation, *IQR* interquartile range, *MPRi* interval based medication possession ratio, *MPRp* prescription based medication possession ratio, *PDC* proportion of days covered

In polytherapy (*n* = 777), the range of adherence rates varied widely, based on whether MPRi or MPRp was used, and also depending on the method used to manage polytherapy (Table 5). The lowest overall estimate of adherence was with using the “low MPRi” method, or taking the lowest class-specific MPRi for each patient (mean 0.47, SD 0.32). The highest overall estimate of adherence was with using the “sum MPRi” method, or by taking the sum of the days supply from each anti-hypertensive medication class as the numerator in the calculation of MPRi (mean 0.95, SD 0.13). “Mean MPRi” (0.66, SD 0.23) and “high MPRi” (0.84, SD 0.21) resulted in adherence estimates between these two extremes. Similarly, when adherence measures were dichotomized, the proportion of adherent users ranged from 24.1% with “low MPRi” to 90.5% with “sum MPRi”. In contrast to patients on monotherapy, PDC and MPRi (regardless of the method used to handle polytherapy) do not give the same adherence estimates for patients on polytherapy.

**Table 5:** Mean and median adherence rates and proportion with adherence ≥0.80 for patients on polytherapy (*n* = 777)

				Range	Mean (SD)	Median (IQR)	Proportion ≥0.80
Overall		MPRi	Low	0.01–1.00	0.47 (0.32)	0.41 (0.16, 0.77)	24.1%
			High	0.15–1.00	0.84 (0.21)	0.93 (0.74, 0.99)	69.6%
			Sum	0.25–1.00	0.95 (0.13)	1.00 (1.00, 1.00)	90.5%
			Mean	0.11–1.00	0.66 (0.23)	0.64 (0.49, 0.87)	33.7%
		MPRp	Low	0.05–1.00	0.84 (0.21)	0.92 (0.77, 0.99)	71.2%
			High	0.10–1.00	0.94 (0.13)	1.00 (0.94, 1.00)	89.5%
			Sum	0.16–1.00	0.96 (0.12)	1.00 (1.00, 1.00)	92.7%
			Mean	0.10–1.00	0.89 (0.15)	0.95 (0.84, 0.99)	81.0%
		PDC		0.15–1.00	0.90 (0.18)	0.98 (0.90, 1.00)	83.0%
Age	65 to 74 years	MPRi	Low	0.01–1.00	0.50 (0.32)	0.48 (0.19, 0.82)	25.2%
			High	0.16–1.00	0.85 (0.19)	0.92 (0.79, 0.99)	73.5%
			Sum	0.33–1.00	0.97 (0.11)	1.00 (1.00, 1.00)	93.2%
			Mean	0.11–1.00	0.67 (0.23)	0.69 (0.49, 0.88)	37.2%
		MPRp	Low	0.05–1.00	0.83 (0.22)	0.91 (0.78, 0.98)	71.8%
			High	0.10–1.00	0.93 (0.15)	1.00 (0.94, 1.00)	89.7%
			Sum	0.20–1.00	0.96 (0.12)	1.00 (1.00, 1.00)	92.7%
			Mean	0.10–1.00	0.88 (0.17)	0.94 (0.84, 0.99)	82.5%
		PDC		0.16–1.00	0.91 (0.16)	0.98 (0.91, 1.00)	85.9%
	75 years and over	MPRi	Low	0.01–1.00	0.46 (0.33)	0.39 (0.16, 0.76)	23.6%
			High	0.15–1.00	0.83 (0.21)	0.94 (0.74, 0.99)	68.0%
			Sum	0.25–1.00	0.95 (0.14)	1.00 (1.00, 1.00)	89.3%
			Mean	0.11–1.00	0.65 (0.23)	0.63 (0.48, 0.87)	32.2%
		MPRp	Low	0.08–1.00	0.84 (0.21)	0.93 (0.77, 0.99)	70.9%
			High	0.20–1.00	0.94 (0.12)	1.00 (0.94, 1.00)	89.4%
			Sum	0.16–1.00	0.96 (0.11)	1.00 (1.00, 1.00)	92.6%
			Mean	0.15–1.00	0.89 (0.15)	0.96 (0.85, 0.99)	80.3%
		PDC		0.15–1.00	0.89 (0.19)	0.99 (0.88, 1.00)	81.8%
Sex	Male	MPRi	Low	0.01–1.00	0.50 (0.33)	0.44 (0.16, 0.82)	26.2%
			High	0.15–1.00	0.83 (0.22)	0.93 (0.74, 0.99)	69.2%
			Sum	0.25–1.00	0.94 (0.15)	1.00 (1.00, 1.00)	89.1%
			Mean	0.11–1.00	0.66 (0.24)	0.67 (0.48, 0.88)	36.6%
		MPRp	Low	0.05–1.00	0.83 (0.22)	0.92 (0.78, 0.98)	70.5%
			High	0.18–1.00	0.93 (0.14)	0.99 (0.93, 1.0)	90.8%
			Sum	0.16–1.00	0.96 (0.13)	1.00 (1.00, 1.00)	91.9%
			Mean	0.15–1.00	0.88 (0.16)	0.95 (0.84, 0.99)	80.9%
		PDC		0.15–1.00	0.88 (0.20)	0.98 (0.85, 1.00)	80.4%
	Female	MPRi	Low	0.01–1.00	0.45 (0.32)	0.38 (0.16, 0.74)	22.3%
			High	0.16–1.00	0.84 (0.19)	0.94 (0.75, 0.99)	70.0%
			Sum	0.33–1.00	0.96 (0.12)	1.00 (1.00, 1.00)	91.6%
			Mean	0.11–1.00	0.65 (0.22)	0.63 (0.49, 0.86)	31.4%
		MPRp	Low	0.10–1.00	0.84 (0.20)	0.93 (0.77, 0.99)	71.7%
			High	0.10–1.00	0.94 (0.13)	1.00 (0.95, 1.00)	88.5%
			Sum	0.20–1.00	0.97 (0.10)	1.00 (1.00, 1.00)	93.3%
			Mean	0.10–1.00	0.89 (0.15)	0.95 (0.85, 0.99)	81.0%
		PDC		0.16–1.00	0.91 (0.16)	0.99 (0.91, 1.00)	85.1%
Income	Lowest quintile	MPRi	Low	0.01–1.00	0.47 (0.31)	0.42 (0.16, 0.75)	20.1%
			High	0.16–1.00	0.83 (0.21)	0.93 (0.74, 0.99)	66.9%
			Sum	0.33–1.00	0.95 (0.14)	1.00 (1.00, 1.00)	90.7%
			Mean	0. 11–1.00	0.65 (0.23)	0.63 (0.46, 0.86)	35.3%
		MPRp	Low	0.10–1.00	0.79 (0.24)	0.89 (0.68, 0.99)	61.6%
			High	0.10–1.00	0.92 (0.16)	1.00 (0.91, 1.00)	85.5%
			Sum	0.16–1.00	0.94 (0.15)	1.00 (1.00, 1.00)	88.5%
			Mean	0.10–1.00	0.86 (0.18)	0.92 (0.80, 0.99)	74.6%
		PDC		0.16–1.00	0.88 (0.19)	0.98 (0.84, 1.00)	79.1%
	Highest quintile	MPRi	Low	0.01–1.00	0.47 (0.32)	0.40 (0.16, 0.79)	24.6%
			High	0.19–1.00	0.83 (0.20)	0.93 (0.69, 0.99)	67.5%
			Sum	0.36–1.00	0.95 (0.12)	1.00 (1.00, 1.00)	90.5%
			Mean	0. 12–1.00	0.65 (0.23)	0.63 (0.49, 0.88)	31.8%
		MPRp	Low	0.14–1.00	0.87 (0.17)	0.94 (0.81, 0.99)	77.6%
			High	0.42–1.00	0.94 (0.11)	1.00 (0.95, 1.00)	92.0%
			Sum	0.35–1.00	0.96 (0.11)	1.00 (1.00, 1.00)	92.9%
			Mean	0.31–1.00	0.91 (0.13)	0.96 (0.87, 0.99)	86.4%
		PDC		0.19–1.00	0.91 (0.17)	0.99 (0.92, 0.99)	86.5%
Charlson comorbidities	0 or 1 comorbidities	MPRi	Low	0.01–1.00	0.46 (0.32)	0.41 (0.16, 0.76)	23.1%
			High	0.15–1.00	0.83 (0.21)	0.93 (0.74, 0.99)	69.1%
			Sum	0.25–1.00	0.95 (0.14)	1.00 (1.00, 1.00)	90.2%
			Mean	0.11–1.00	0.65 (0.23)	0.63 (0.48, 0.87)	32.3%
		MPRp	Low	0.05–1.00	0.83 (0.21)	0.92 (0.77, 0.99)	71.1%
			High	0.10–1.00	0.94 (0.13)	1.00 (0.94, 1.00)	89.5%
			Sum	0.16–1.00	0.96 (0.12)	1.00 (1.00, 1.00)	92.7%
			Mean	0.10–1.00	0.89 (0.16)	0.95 (0.84, 0.99)	80.5%
		PDC		0.15–1.00	0.90 (0.18)	0.98 (0.90, 1.00)	83.1%
	2+ comorbidities	MPRi	Low	0.05–1.00	0.54 (0.34)	0.57 (0.16, 0.85)	33.3%
			High	0.25–1.00	0.86 (0.19)	0.96 (0.80, 1.00)	74.7%
			Sum	0.41–1.00	0.97 (0.10)	1.00 (1.00, 1.00)	93.3%
			Mean	0.11–1.00	0.71 (0.24)	0.76 (0.51, 0.92)	46.7%
		MPRp	Low	0.08–1.00	0.85 (0.19)	0.93 (0.75, 0.99)	72.0%
			High	0.48–1.00	0.94 (0.12)	1.00 (0.96, 1.00)	89.3%
			Sum	0.53–1.00	0.97 (0.09)	1.00 (1.00, 1.00)	92.0%
			Mean	0.36–1.00	0.90 (0.13)	0.95 (0.85, 0.99)	85.3%
		PDC		0.25–1.00	0.91 (0.15)	0.99 (0.88, 1.00)	82.7%

*Abbreviations*: *SD* standard deviation, *IQR* interquartile range, *MPRi* interval based medication possession ratio, *MPRp* prescription based medication possession ratio, *PDC* proportion of days covered

The range of adherence was much narrower for MPRp adherence estimates, with “low MPRp” giving again the lowest adherence estimate (mean 0.84, SD 0.21) and “sum MPRp” giving the highest estimate (mean 0.96, SD 0.12). The variation in the proportion of adherent users was also much smaller using MPRp, ranging from 71.2% with “low MPRp” to 92.7% with “sum MPRp”. PDC gave a more conservative measure compared to both sum MPRi and sum MPRp; 83.0% of the sample was classified as adherent using PDC. Even with stratification across age, sex, income, and comorbidities, the same patterns could be seen where MPRi’s gave much wider ranges of adherence estimates compared with MPRp’s, with “low MPR” giving the lowest estimates and “sum MPR” giving the highest estimates regardless of whether the prescription or interval based MPR method was used.

### Association between adherence and mortality

A total of 387 (17.6%) deaths occurred in the follow-up period. Greater medication adherence was consistently associated with lower odds of death, after risk adjustment (see Table 6). Overall, the strength of association was stronger for MPRp than for MPRi or PDC (adjusted odds ratio [aOR] for “sum MPRp” was 0.70 [95% CI 0.51, 0.97] for “sum MPRi” aOR 0.78 [95% CI 0.58, 1.05], for PDC aOR 0.80 [95% CI 0.60, 1.07]). Of the numerous methods of handling concurrent medication use, “sum MPRp” and “mean MPRp” had the strongest associations with mortality with these being the only measures to reach statistical significance (aOR 0.70 [95% CI 0.51, 0.97] and 0.71 [95% CI 0.53, 0.95] respectively).

**Table 6:** Adjusted logistic regression modeling the odds ratio of death, for patients who are adherent versus non-adherent to anti-hypertensive medication

			Adjusted OR^b^	95% CI	*P* value
Overall	MPRi	Low	0.96	(0.74, 1.23)	0.743
		High	0.90	(0.68, 1.19)	0.475
		Sum	0.78	(0.58, 1.05)	0.104
		Mean	0.88	(0.68, 1.13)	0.321
	MPRp	Low	0.79	(0.59, 1.05)	0.098
		High	0.76	(0.56, 1.05)	0.094
		Sum	0.70	(0.51, 0.97)	0.029
		Mean	0.71	(0.53, 0.95)	0.021
	PDC		0.80	(0.60, 1.07)	0.130
Monotherapy	MPRi		0.80	(0.40, 1.57)	0.510
	MPRp		0.74	(0.33, 1.66)	0.461
	PDC		0.80	(0.40, 1.57)	0.510
Polytherapy	MPRi	Low	1.06	(0.64, 1.73)	0.832
		High	1.15	(0.71, 1.85)	0.578
		Sum	0.74	(0.37, 1.49)	0.400
		Mean	0.83	(0.53, 1.31)	0.426
	MPRp	Low	0.84	(0.53, 1.33)	0.455
		High	0.99	(0.50, 1.97)	0.975
		Sum	0.65	(0.31, 1.36)	0.251
		Mean	0.66	(0.40, 1.10)	0.112
	PDC		0.80	(0.46, 1.38)	0.424

^b^ Adjusted for age, sex, Charlson comorbidity index, income quintile, and previous hospitalization for cardiovascular disease

Where “Low” = adherence estimate for the single medication class with the lowest adherence; “High” = adherence estimate for the single medication class with the highest adherence; “Sum” = adherence to *any* medication class (where days supply for each medication class summed as the numerator); “Mean” = mean adherence to each medication class

*Abbreviations*: *OR* odds ratio, *CI* confidence interval, *MPRi* interval based medication possession ratio, *MPRp* prescription based medication possession ratio, *PDC* proportion of days covered

Similarly, in monotherapy, the association between medication adherence and mortality was stronger for MPRp measures than for MPRi and PDC measures based on point estimates, despite none reaching statistical significance. In polytherapy patients, the same inverse association could be seen between medication adherence as measured by MPRp and death, with the strongest associations seen again with “sum” and “mean” MPRp, though again none reached statistical significance.

Unadjusted and adjusted Cox proportional hazards regression provided similar results to logistic regression. However, testing of the proportional hazards assumption indicated its violation. Kaplan-Meier survival curves revealed a divergence of survival in adherent versus non-adherent groups at approximately 3.5 to 4 years post index date of hypertension diagnosis for MPRp definitions (with a less prominent trend using MPRi and PDC definitions).

### Sensitivity analysis

Similar results were seen when adjusted logistic regression was performed using different adherence thresholds for each of the operational definitions (Appendix 2). Across all adherence thresholds, adherence estimates as measured by MPRp were more strongly associated with mortality than as measured by MPRi. The MPRp definitions that showed a statistically significant association between adherence and mortality varied depending on the adherence threshold used. Statistical significance for this association was reached for mean MPRp when using adherence thresholds of 0.70, 0.75, and 0.80; for sum MPRp, the adherence thresholds were 0.80 and 0.85. For low MPRp, statistical significance was reached when the adherence threshold used was 0.70.

## Discussion

In a cohort of 2199 patients with incident hypertension, we found that different definitions of medication adherence resulted not only in different baseline characteristics of “adherent” and “non-adherent” groups but also vast differences in estimated adherence rates. Similar to previous studies, adherence measures based on a fixed observation period (MPRi and PDC) resulted in lower adherence estimates compared with variable observation periods based on prescription refill dates (MPRp) [8, 21]. In addition, different methods of calculating adherence for patients on polytherapy provided very different estimates of adherence [8]. For example, if only those with an MPRi of ≥0.80 for each and every medication class were considered adherent, only 24.1% would be classified as being adherent. Conversely, if we considered those with an overall MPRi of ≥0.80, when all medication classes were grouped together, as adherent, over 90% of the same sample population would be classified as adherent. Therefore, given this wide variation in adherence estimates and their implications on study conclusions, it is imperative that future adherence studies are transparent in providing information regarding: 1) specific operational definitions of adherence used and the numerator and denominator used in these calculations; and 2) the method used to manage polytherapy. Because adherence measure names and corresponding calculations are inconsistent across studies [7], we note that adherence measure terms alone are inadequate in describing the differences in calculations.

Adjusted logistic regression revealed that regardless of the operational definition used for medication adherence, there was a non-statistically significant trend between adherence to anti-hypertensive medications and lower risk of death. The strength of association was greater in prescription-based measures compared with interval-based measures. However, both methods are not without their faults. While prescription-based measures likely overestimate adherence by not accounting for patients who inappropriately discontinue their medications [22], interval-based measures likely underestimate adherence, by interpreting medication switches as non-adherence.

Our sensitivity analysis revealed that the operational definitions that demonstrate the strongest and most significant associations between adherence and mortality vary depending upon the adherence threshold used. When using low MPRp, adherence above the threshold of 0.70 is significantly associated with lower risk of mortality; this significance is lost when higher thresholds are used. For mean MPRp measures, adherence using the thresholds of 0.70 to 0.80 is associated with reduced risk of death, but not when using higher thresholds of 0.85 or 0.90. For sum MPRp, adherence above the threshold of 0.80 and 0.85 is associated with reduced risk of death. Therefore, the optimal adherence threshold may differ based on the method used to handle polytherapy. When using stringent measures of adherence (such as when adherence is measured based on the medication with lowest adherence in the case of low MPRp), a lower adherence threshold such as 0.70 may be preferred. In contrast, when using a more liberal measure of adherence (such as considering a patient adherent even if he or she adheres to only one of numerous medications, such as in the case of sum MPRp), a higher adherence threshold such as 0.80 or 0.85 may be preferred.

For the standard adherence threshold of 0.80, the adherence-mortality association reached statistical significance only when using mean and sum prescription-based MPR measures. These measures likely reflect “overall” adherence to a patient’s full regimen of anti-hypertensive medications. Being adherent to only one medication and non-adherent to others (that is, taking into account only highest MPR) is not significantly associated with reduced risk of death, as it may not reflect global adherence to all medications. In contrast, adherence based on “low MPR” (that is, all medication classes must have adherence ≥0.80) is likely too stringent a criterion, and its high specificity would result in many patients being considered non-adherent. The mean and sum MPRp are likely a balance between these two extremes, where non-adherence to at least one medication class in a regimen can profoundly affect these adherence estimates. Our study suggests that mean and sum MPRp are the preferred operational measures of adherence, when using the adherence threshold of 0.80, given their significant association with mortality.

Certain limitations in our study deserve consideration. First, to allow calculation of medication adherence over a one-year observation period, we excluded patients who died or did not have at least one prescription refill within one year of their first prescription fill. We have therefore likely excluded those who are sickest and most dependent on chronic medication therapy, and those who are most non-adherent. As a result, adherence is likely overestimated and the association between adherence and mortality weakened. Second, a maximum of five years of follow-up may be insufficiently long to assess association between adherence and mortality. This concern is supported by Kaplan Meier survival curves showing divergence for the non-adherent and adherent groups beginning only at 3.5 to 4 years from index date of diagnosis. Third, it is possible that the older age of the sample population could have confounded the association between medication adherence and mortality. A Cochrane systematic review showed that in the elderly aged 80 years or older, anti-hypertensive therapy did not reduce total mortality, though it did reduce cardiovascular mortality and morbidity [23]. Because 25% of our sample population is aged 80 years or older, in whom there may not be mortality benefit of anti-hypertensive therapy, the overall associations between anti-hypertensive adherence and mortality may have been weakened by this subgroup. Fourth, because we did not directly compare the different operational definitions of adherence, we cannot state the superiority of one definition over another and therefore cannot recommend a single standardized operational definition for adherence. Rather, our findings suggest that different operational definitions of adherence result in very different estimates of adherence, and certain definitions are associated with long-term outcomes in patients with hypertension, while others are not. Lastly, we used adherence thresholds of 0.80 and a one-year observation period due to its wide usage throughout the literature, despite limited evidence to support these parameters. However, our sensitivity analysis shows similar results and conclusions when using varying adherence thresholds. We recommend confirmation of our findings in a separate large cohort of patients with hypertension, to ensure the validity and generalizability of our conclusions.

## Conclusion

This study is an important contribution to the literature, by providing a better understanding of commonly used medication adherence definitions and their association with outcomes. We recommend the use of prescription-based denominators when calculating medication possession ratios and related measures given their narrower range of estimates and stronger associations with long-term outcomes. In patients using concurrent medications, we recommend using the methods of handling polytherapy that are most strongly associated with mortality. For a standard adherence threshold of 0.80, these include the mean medication possession ratio (measured as an average of individual class-specific MPRs) and “sum” medication possession ratio (measured as adherence to *any* medications in the relevant medication classes). We have shown clearly that different operational definitions of medication adherence and different methods of handling polytherapy can result in a wide range of adherence estimates and therefore conclusions reached about adherence, highlighting the need to harmonize these definitions. Given these implications, it is of utmost importance that future adherence studies using pharmacy claims data carefully select and describe the medication adherence definitions used, especially for patients using multiple concurrent medications.

## Appendix 1

**Table 7:** Antihypertensive medication classes

Medication Class	Medication Name
Thiazide-like Diuretic	Bendroflumethiazide Chlorthalidone Hydrochlorothiazide Indapamide Metolazone
Angiotensin Converting Enzyme Inhibitors *or* Angiotensin Receptor Blockers	Benazepril Captopril Cilazapril Enalapril Fosinopril Lisinopril Perindopril Quinapril Ramipril Trandolapril Candesartan Eprosartan Irbesartan Losartan Telmisartan Valsartan
Beta Blockers	Acebutolol Atenolol Bisoprolol Labetalol Metoprolol Nadolol Oxprenolol Pindolol Propranolol Timolol
Calcium Channel Blockers	Amlodipine Diltiazem Felodipine Nicardipine Nifedipine Verapamil
Combination containing at least one of the above medication classes	Atenolol + Diuretics Candesartan + Diuretics Cilazapril + Diuretics Enalapril + Diuretics Eprosartan + Diuretics Hydrochlorothiazide + Potassium Sparing Agents Irbesartan + Diuretics Lisinopril + Diuretics Losartan + Diuretics Methyldopa + Diuretics Nadolol + Diuretics Perindopril + Diuretics Pindolol + Diuretics Propranolol + Diuretics Quinapril + Diuretics Reserpine + Diuretics Telmisartan + Diuretics Timolol + Diuretics Valsartan + Diuretics

## Appendix 2

**Table 8:** Sensitivity analysis for odds ratios modeling death, for patients who are adherent versus non-adherent to anti-hypertensive medication, using different thresholds to define adherence

			Adjusted OR^a^	95% CI	P value
Adherence >0.65	MPRi	Low	0.96	(0.74, 1.26)	0.787
		High	0.91	(0.65, 1.26)	0.560
		Sum	0.89	(0.62, 1.27)	0.509
		Mean	0.99	(0.75, 1.31)	0.940
	MPRp	Low	0.74	(0.52, 1.06)	0.101
		High	0.81	(0.53, 1.23)	0.317
		Sum	0.80	(0.52, 1.22)	0.294
		Mean	0.78	(0.53, 1.16)	0.222
	PDC		0.84	(0.60, 1.14)	0.323
Adherence >0.70	MPRi	Low	0.92	(0.71, 1.19)	0.514
		High	0.87	(0.64, 1.19)	0.385
		Sum	0.78	(0.56, 1.09)	0.151
		Mean	0.88	(0.67, 1.14)	0.324
	MPRp	Low	0.71	(0.51, 0.98)	0.036
		High	0.71	(0.49, 1.02)	0.063
		Sum	0.69	(0.48, 1.00)	0.052
		Mean	0.64	(0.45, 0.90)	0.011
	PDC		0.77	(0.56, 1.07)	0.117
Adherence >0.75	MPRi	Low	0.96	(0.75, 1.24)	0.767
		High	0.91	(0.68, 1.21)	0.515
		Sum	0.81	(0.59, 1.11)	0.186
		Mean	0.88	(0.68, 1.14)	0.322
	MPRp	Low	0.74	(0.55, 1.00)	0.053
		High	0.77	(0.54, 1.08)	0.132
		Sum	0.71	(0.50, 1.00)	0.052
		Mean	0.70	(0.51, 0.97)	0.030
	PDC		0.82	(0.61, 1.11)	0.203
Adherence >0.80	MPRi	Low	0.96	(0.74, 1.23)	0.743
		High	0.90	(0.68, 1.19)	0.475
		Sum	0.78	(0.58, 1.05)	0.104
		Mean	0.88	(0.68, 1.13)	0.321
	MPRp	Low	0.79	(0.59, 1.05)	0.098
		High	0.76	(0.56, 1.05)	0.094
		Sum	0.70	(0.51, 0.97)	0.029
		Mean	0.71	(0.53, 0.95)	0.021
	PDC		0.80	(0.60, 1.07)	0.130
Adherence >0.85	MPRi	Low	0.89	(0.69, 1.15)	0.367
		High	0.83	(0.64, 1.08)	0.175
		Sum	0.75	(0.57, 0.99)	0.044
		Mean	0.88	(0.69, 1.13)	0.329
	MPRp	Low	0.80	(0.61, 1.05)	0.103
		High	0.80	(0.59, 1.07)	0.136
		Sum	0.70	(0.52, 0.94)	0.018
		Mean	0.80	(0.61, 1.06)	0.120
	PDC		0.79	(0.60, 1.04)	0.095
Adherence >0.90	MPRi	Low	0.95	(0.74, 1.23)	0.713
		High	0.92	(0.71, 1.18)	0.500
		Sum	0.85	(0.65, 1.12)	0.244
		Mean	0.93	(0.72, 1.20)	0.589
	MPRp	Low	0.83	(0.64, 1.07)	0.156
		High	0.89	(0.68, 1.18)	0.427
		Sum	0.79	(0.59, 1.04)	0.094
		Mean	0.86	(0.66, 1.11)	0.250
	PDC		0.88	(0.67, 1.14)	0.332

^a^ Adjusted for age, sex, Charlson comorbidity index, income quintile, and previous hospitalization for cardiovascular disease

Where “Low” = adherence estimate for the single medication class with the lowest adherence; “High” = adherence estimate for the single medication class with the highest adherence; “Sum” = adherence to *any* medication class (where days supply for each medication class summed as the numerator); “Mean” = mean adherence to each medication class

*Abbreviations*: *OR* odds ratio, *CI* confidence interval, *MPRi* interval based medication possession ratio, *MPRp* prescription based medication possession ratio, *PDC* proportion of days covered

## Abbreviations

ACEI: Angiotensin converting enzyme inhibitor
aOR: Adjusted odds ratio
ARB: Angiotensin receptor blocker
BB: Beta blocker
CCB: Calcium channel blocker
CI: Confidence interval
ICD-10: International Classification of Diseases, Tenth Revision
ICD-9: International Classification of Diseases, Ninth Revision
MPR: Medication possession ratio
MPRi: Interval based medication possession ratio
MPRp: Prescription based medication possession ratio
OR: Odds ratio
PDC: Proportion of days covered
